# Supplementary material for: The fly connectome reveals a path to the effectome
Source: Nature. 2024 Oct 2;634(8032):201–9. doi: 10.1038/s41586-024-07982-0 (PMC11446844; doi:10.1038/s41586-024-07982-0)
Supplement: Supplementary file 1 — This file has five sections: (1) Graphical model of IV estimator; (2) Experimental approach; (3) IV estimator applied to nonlinear dynamics; (4) IV estimator additional simulations; and (5) Additional connectome analyses. [file 41586_2024_7982_MOESM1_ESM.pdf]

nature portfolio

https://doi.org/10.1038/s41586-024-07982-0

Supplementary information

The fly connectome reveals a path to the effectome

In the format provided by the authors and unedited

Supplementary Information

Graphical model of IV estimator

The graphical model associated with our idealized experimental setup is depicted Extended Data Fig. 1. All variables are vectors and parameters are matrices. Dark arrows indicate estimands, grey arrows and variables respectively indicate parameters that cannot be estimated and variables that cannot be observed. Z_t are unobserved neurons that potentially interact with observed populations. A critical assumption is that optogenetic perturbation influences source neurons before it affects target neurons (L_t direct arrow in to X_t) and do not directly affect other neurons or neural activity at other time points. In this model only neural activity in the previous time step directly effects neural activity in the next time step (arrows into X_t, Y_t, Z_t only from X_{t-1}, Y_{t-1}, Z_{t-1}). Note the similarity of the graph structure in Fig. 1B and here: for example the linear relationship between X_{t-1} and Y_t is the causal effect of interest and Y_{t-1} and Z_{t-1} are confounders. This approach can be extended to an VAR(p) model to account for differing delays between neurons.

Experimental approach **(proposal by M. Aragon and D. Pospisil)**

Our simulations in the main text focused on the stimulation of a single neuron while the entire fly brain is observed. A conceptually straight-forward approach to learning the entire effectome would be to stimulate one of each of the 121,327 fly neurons in 121,327 different flies while imaging the whole-brain. This amounts to learning one column of the causal effect matrix per a fly (Extended Data Fig. 2A). Yet performing this number of experiments would be difficult.

Learning the effectome more rapidly requires a higher rank perturbation. In the case of optogenetics, the rank could be increased with 2-photon holographic stimulation so that arbitrary sets of neurons could receive some degree of independent drive [46]. If every neuron in the fly brain was stimulated independently while every neuron was observed, the entire fly effectome could be identified within a single experiment (Extended Data Fig. 2B).

A common issue with the two hypothetical approaches described above is the difficulty of observing all neurons simultaneously. Resolving single neurons from densely labeled neuropil recordings is an open problem. Similarly, independently targeting all neurons for whole-brain patterned stimulation is infeasible given the constraints of diffraction-limited optics. Moreover, the amount of energy required to optogenetically target all neurons in the brain would likely heat the brain tissue and interfere with its function. Here, we propose an alternative approach to learning the effectome that could be achieved in a feasible number of experiments, permits accurate identification of neurons, and leverages existing technologies.

Learning the effectome incrementally

We now turn to a strategy of learning the entire fly effectome using Bayes-IV. Our goal is to learn the effectome using genetically identical flies under the same experimental conditions (e.g., at the onset of a stimulus). For example the effectome could be measured during optic flow stimuli to gain insight into whole-brain neural dynamics during visual motion.

We propose that sparse populations of neurons should express both opsin and a voltage sensor so that they can be both stimulated and observed simultaneously. Holographic optogenetic stimulation can then be used to drive each neuron independently and the voltage sensor can report the effect. Recently developed experimental techniques allow recording from the fly brain for up to 12 hours [47]: this would allow for a block of the effectome weights for a complete subset of neurons to be learned over the course of a single experiment (Extended Data Fig. 2C, see Sparse expression for further detail).

The efficiency of this approach depends on the size of the population that expresses both opsin and an voltage sensor (source population). To evaluate efficiency, we consider percent identifiability as a function of number of experiments per neurons total. Percent identifiability is simply the percent of the effectome that could be learned given an unlimited amount of data (i.e., consistency for a portion of of the effectome matrix). We consider this in units of experiments per neuron because for a given application the investigator may want to learn the effectome of a sub-population of neurons.

For identifiability of all post-synaptic weights for n_S source neurons and a perturbation of rank n_l, at least $\frac{n_S}{n_L}$ experiments are needed to identify the post-synaptic weights on n_T target neurons. Thus to identify the weights on all D post-synaptic neurons will require repeating this procedure at least $\frac{N}{n_T}$ times. So overall the number of experiments needed to identify all of the source neurons post-synaptic weights ($n_{E(S)}$) is,

$$n_{E(S)} = \frac{n_S N}{n_L n_T}. \tag{1}$$

Then the total number of experiments (N_E) to identify the entire effectome, assuming the number of source neurons is the same for each experiment is simply Supplementary Information Equation 1 multiplied by $\frac{N}{n_S}$,

$$N_E = \frac{N^2}{n_L n_T}, \tag{2}$$

at worst this is N^2 and at best 1.

For reference, we first considered the exhaustive approach of stimulating each neuron individually (Extended Data Fig. 2A). In this case, the effectome would accumulate slowly and require the same number of experiments as neurons (D, thick red trace intersects 100 % at 1). In general with a perturbation rank of 1, at least as many experiments as neurons would be needed. On the other hand, our sparse patterned stimulation approach achieves full identifiability with orders of magnitude greater efficiency, even if only 5 % of neurons are observed for each experiment (medium green trace intersects 100% at 420 experiments). This rate could be achieved even more rapidly with higher expression rates (thicker green trace) but at the cost of increased

difficulty of identifying and stimulating larger populations of neurons. Thus even if only small subsets of the brain could be simultaneously observed during patterned perturbations the effectome could be feasibly estimated.

Experimental setting

Identifiability does not guarantee an accurate estimate of the weights but is necessary for a consistent estimate. The amount of data needed to achieve an accurate estimate is a result of factors within single experiments, principally the SNR of the imaging modality, the strength of perturbation, and the number of samples. These factors are unknown in many of the experimental setups we recommend as they would vary from neuron-to-neuron, perturbation technique, and imaging technique. It seems plausible that within a single experiment enough samples could be collected to account for these other factors given recent approaches that allow up to 12 hours of continuous recording of neural activity in the fly [47].

Here we specify the sufficient, feasible experimental setting to estimate the effectome given sufficient SNR. In brief, if these requirements are satisfied we would be able to measure the direct effects of perturbations on the voltage of stimulated neurons and the subsequent effects on downstream neurons after a conduction delay with each neurons being identified with the connectome.

(1) Sparse expression

We propose expressing voltage sensors and opsin in sparse subpopulations of neurons. Specifically, these neurons should correspond to those with the highest loadings in each of the eigenvectors obtained through eigendecomposition of the connectome prior (see Results, 'Global dynamical properties of the putative effectome'). This prior can of course be updated as experiments progress. In the case where the neurons of interest have highly interdigitated neurites, segmenting these neurons for downstream identification may be infeasible. To address this, driver lines used may be further sparsened using the Sparse Predictive Activity through Recombinase Competition (SPARC) genetic toolkit [48]. SPARC will enable simultaneous expression of opsin and voltage sensor in the same sparsened subpopulation of neurons, which is required by the approach we have outlined.

We acknowledge that using a single expression system, such as GAL4-UAS, may be an appropriate strategy for only a subset of eigencircuits. Necessarily, neurons with expression in a given driver line must share a genetic lineage. However, non-localized eigencircuits may contain neurons from diverse lineages. One strategy for addressing this limitation is to employ multiple binary expression systems in parallel, including GAL4-UAS, LexA-LexAoP, and QF/QUAS. Together, these orthogonal expression systems may permit broader expression of the voltage sensor and opsin while preserving sparsity. This approach remains to be tested in the context of our proposed experimental setting, but we remain optimistic that the current genetic tools available in Drosophila neuroscience offer a promising starting point for interrogating the putative eigencircuits of the fly brain.

Here we provide a concrete example of selecting GAL4 driver lines based on the putative effectome. Eigenvector 25 contains neurons within the antennal lobe, mushroom body, and lateral horn (Fig. 3G bottom center); the spatial segregation of these regions makes this circuit amenable to our proposed experimental strategy. To begin, we determine the neurons in this circuit with the highest loadings: these neurons include DP1m-adPN, v2LN30, VL2p-adPN, and DP1l-adPN. Next, we find candidate GAL4 lines that contain hits for these neurons of interest using publicly available resources [21]. We find that the GAL4 line VT014336 contains strong pixel overlap scores for each of these neurons, and is thus a suitable candidate for uncovering the causal interactions of neurons within eigenvector 25.

(2) Population voltage recordings

Our estimator also requires rapid imaging speeds to uncover causal effects. In the most conservative case the time difference over which IV estimates are calculated would be the conduction delay of action potentials between the neurons being observed (1 ms conduction times typical [49]). Fortunately, the top neurons within each eigenvector are often anatomically localized, potentially meaning that only small sub-volumes of the brain need to be scanned during any given experiment. Nevertheless, the current acquisition speed available on most consumer 2-photon microscopes may still be insufficient to capture neural dynamics at the fastest timescales required to estimate a direct causal effect between neurons. Although custom microscopes have been engineered to achieve kilohertz-rate imaging [50], making this technology widely accessible to *Drosophila* neuroscientists will be a crucial step toward a community-driven effort to accurately estimate an effectome for the entire fly brain. Leveraging the imaging speed offered by these technologies will require activity indicators with the fastest possible kinetics. Voltage indicators like ASAP1 achieve depolarization and re-polarization dynamics on the order of 1 ms [51], and have been successfully used to record activity in the fly brain [52].

(3) Patterned optogenetic stimulation

Estimation efficiency of the IV estimator increases as a function of effectively independent stimulation channels. To this end, we propose independently stimulating each labeled neuron in the brain using 2-photon holographic stimulation (HS). Although 2-photon HS has been used in other animal systems, most notably mice, to our knowledge no work has attempted to extend this technology to flies. This technological lag may be partly due to the fact that genetic drivers in the fly can result in exceptionally sparse labeling of neurons. As a result, probing specific neural circuits with optogenetics can be achieved through genetic, rather than optical, precision. However, in our framework both genetic and optical control are required to learn the effectome, thus necessitating the introduction of holography to the fly. HS is a mature technology that can generate arbitrary light patterns in 3D volumes using spatial light modulators (SLMs). Multiple SLMs used in parallel can generate kilohertz-rate stimulation across approximately $1mm^3$ volumes [53], which is larger than the volume of the adult *Drosophila melanogaster* brain. This rapid stimulation rate over large volumes will

be complemented by state-of-the-art opsins like Chronos, which achieves millisecond-timescale on and off-kinetics and is available for the fly [54]. Crucially, the sparse labeling outlined above will reduce the total laser power required to drive activity across the labeled population, thus preserving the health of the imaged fly.

(4) Neural identification

The last major technical hurdle toward estimating the effectome involves matching neurons that were recorded and perturbed experimentally with neurons in the connectome. Once again, the sparsity of each putative effectome circuit greatly facilitates this matching problem. Furthermore, recent work has successfully demonstrated registration of single cell types imaged at light level to the connectome [55]. However, whether existing registration approaches can unambiguously assign single neuron identities to each neuron in the experimental population remains untested. We provide a detailed strategy below.

Creating the effectome for the fly brain requires mapping single-neuron dynamics to identified neurons. This is now possible with the whole-brain connectome [1] associated with the Full Adult Female Brain (FAFB) electron microscopy dataset [4]. To accomplish this, we propose following the BrIdge For Registering Over Statistical Templates (BIFROST) registration pipeline [55]. In short, the experimental fly will express a pan-neuronal structural marker (td-tomato) in addition to the opsin and voltage sensor under control of the sparse genetic driver. This structure channel data (tomato-fast) will be acquired simultaneously with the functional voltage sensor data. After the experiment is complete, a high-resolution scan of the voltage sensor channel and td-tomato channel (tomato-slow) will be acquired. The high resolution scans acquired across experiments will be used to generate a mean brain template. Registration to the FAFB space is then accomplished through the following transformations: (a) tomato-fast to tomato-slow; (b) tomato-slow to mean brain; (c) mean brain to the standard functional *Drosophila* atlas (FDA); (d) FDA to FAFB. The first three steps rely on linear and nonlinear transformations, which can be accomplished using the ANTs library [56]. The final transformation is accomplished using a neural network called SynthMorph [57], which is able to bridge the disparate image statistics of the FDA and FAFB spaces. Finally, the transformations for the structural data will be applied to the functional voltage sensor channel.

The final step in our proposed experimental framework is to assign unique IDs to each neuron that expresses opsin and voltage sensor. To do this, we suggest a two-pronged approach. First, the spatial footprint of ROIs extracted from the functional channel will be registered to the FAFB space, as outlined above. We expect that the ROIs may not perfectly align with the neural skeletons in the FAFB dataset. To address this, single-neuron light-level skeletons may be generated from the high-resolution anatomical scan obtained at the end of each experiment. NBLAST, an algorithm that computes anatomical similarities across datasets [58], may then be used to find candidate neurons in the FAFB datset with high anatomical similarity to the light-level neural skeleton that overlaps with a particular ROI. Critically, the location of the registered ROI in the FAFB space constrains the set of candidate neurons with which to perform NBLAST, thus greatly enhancing the efficiency of this process. The

final neuron ID will then be determined by taking the consensus between the ROI registration approach and the top NBLAST candidates.

It is difficult to predict how this particular experimental approach will perform in any given circuit or experimental condition without actually attempting it. This approach can at minimum serve as a starting point that uses existing technologies and upon it, if needed, iterative technological enhancements can be developed to estimate the effectome.

IV estimator applied to nonlinear dynamics

Discrete VAR(1) nonlinear dynamics

Our estimator was derived under the assumption that effects between neurons over time are determined by a linear dynamical system. However, real neuronal networks are known to form a nonlinear dynamical systems. Thus, it is important to carefully examine how our method performs when applied to a realistic nonlinear dynamical model of neural activity.

To address this problem, we begin by considering a discrete-time recurrent neural network (RNN) of the following form:

$$\mathbf{r}_t = f(W\mathbf{r}_{t-1} + W_{l,x}L_t + \epsilon_t) \tag{3}$$

where $f(\cdot)$ is a nonlinearity applied individually each neuron at each time step.

We consider the principle categorical benefit of using the IV estimator in the linear case: it does not mistake unobserved common inputs for causal effects. We find that this property is preserved when the linear estimator is applied to the nonlinear dynamics described above but under more stringent conditions. Specifically we require that $W_{l,x}$ is an invertible diagonal matrix (each neuron is independently stimulated) and we can observe subthreshold activity (before the nonlinearity is applied) then this property holds for the nonlinear case. This can be seen by considering the parameters the IV estimator converges to in the nonlinear case:

$$\begin{aligned}&\mathrm{Cov}[Y'_{t+1}, L_t](\mathrm{Cov}[X'_t, L_t])^{-1}\\&= W_{x,y}\mathrm{Cov}[f(W_{l,x}L_t + W_x X_{t-1} + W_{y,x}Y_{t-1} + W_{z,x}Z_{t-1} + \epsilon_t), L_t]W_{l,x}^{-1}\end{aligned} \tag{4}$$

where Y'_t and X'_t are sub-threshold activity. By assumption we know that $W_{l,x}$ is diagonal thus its inverse is also diagonal and we know the middle covariance factor is diagonal because,

$$\begin{aligned}&\mathrm{Cov}[f(W_{l,x}L_t + W_x X_{t-1} + W_{y,x}Y_{t-1} + W_{z,x}Z_{t-1} + \epsilon_t), L_t]_{i,j}\\&= \mathrm{Cov}[f(W_{l,x,i,i}L_{t,i} + W_x X_{t-1,i} + W_{y,x}Y_{t-1,i} + W_{z,x}Z_{t-1,i} + \epsilon_{t,i}), L_{t,j}]\end{aligned} \tag{5}$$

and when $i \neq j$ there is no dependence between any random variables and thus covariance is 0. Thus in our estimator we are left with a diagonal matrix which rescales the $W_{x,y}$ but any entries that are 0 remain 0. Conversely, if the stimulation is not

independently applied to each neuron there is no guarantee this property will be maintained. We must then rely on $f(\cdot)$ being effectively linear in the range of subthreshold voltages.

Conductance-based neural dynamics model

Here we consider the interpretation of the IV estimator in the context of a nonlinear neural dynamics model. In summary, we find the linear IV estimator converges to the Jacobian of the neural dynamics model–often used as a linear approximation to nonlinear dynamics. We find the Jacobian and the connectome are only guaranteed to be equal when there is no synapse between neurons. In simulation we find this is sufficient to provide large gains in statistical efficiency in the context of sparse connectomes.

We employ a classic nonlinear neural dynamics model for which there is both a nonlinearity induced by neuronal firing rate's relationships to voltage and the multiplicative dependence of synaptic currents on membrane voltage [44]. Voltage dynamics are governed by the following equation:

$$\tau \frac{\partial \mathbf{v}_m(t)}{\partial t} = R\mathbf{W}_0 \odot (\mathbf{E} - \mathbf{v}_m(t)1_D^T) f(\mathbf{v}_m(t-\mathbf{d})) + \mathbf{v}_{\text{rest}} - \mathbf{v}_m(t), \tag{6}$$

where $\mathbf{v}_m(t)$ is the $D \times 1$ vector of membrane voltages at time t, $\mathbf{W}_0$ is the $D \times D$ matrix of post-synaptic conductance (i.e., for a unit firing rate in the pre-synaptic neuron the change in conductance in the post-synaptic site–often simplified to the product of probability of pre-synaptic release, the number of pre-synaptic NT release sites and the peak conductivity per vesicle release), $\mathbf{E}$ is the $D \times D$ matrix of receptor specific reversal potentials (this determines whether a synapse is inhibitory, excitatory, or shunting), $f(\cdot)$ is the firing rate (here a sigmoid), $\mathbf{d}$ is a vector of conduction delays, and $\mathbf{v}_{\text{rest}}$ is the $D \times 1$ vector of resting membrane potentials.

We note that it is typical to approximate nonlinear dynamics with a Jacobian linearization around an equilibrium point of the dynamics—a value $\bar{\mathbf{v}}_m$ where $\left.\frac{\partial \mathbf{v}_m(t)}{\partial t}\right|_{\mathbf{v}_m = \bar{\mathbf{v}}_m} = 0$. Specifically, letting $\delta = \mathbf{v}_m(t) - \bar{\mathbf{v}}_m$ and $\mathbf{J} := \left.\frac{\partial f}{\partial \mathbf{v}_m}\right|_{\mathbf{v}_m = \bar{\mathbf{v}}_m}$ where $f = \tau \frac{\partial \mathbf{v}_m(t)}{\partial t}$ (Supplementary Information Equation 6) then,

$$\frac{\partial}{\partial t}\delta(t) = \mathbf{J}\delta(t) \tag{7}$$

provides a linear dynamical system that approximates the original nonlinear dynamics to arbitrary precision as variation about the about the equilibrium point decreases. In the specific case of the conductance model (ignoring the conduction delay and just considering off-diagonals of the Jacobian for clarity),

$$\mathbf{J} = \tau R\mathbf{W}_0 \odot \left.\frac{\partial}{\partial v_m}(\mathbf{E} - v_m 1_D^T) f(v_m)\right|_{\mathbf{v}_m = \bar{\mathbf{v}}_m} - \tau I. \tag{8}$$

Given that the IV estimator, for weak enough perturbations, estimates the linear effects of local perturbations, we investigated whether it consistently estimated the

Jacobian of the conductance model in simulations (Extended Data Fig. 3). We first examined the case where dynamics were stable and the average voltage across neurons was similar (Extended Data Fig. 3C). In this case, the IV estimator converged to the Jacobian of the conductance model evaluated at the average voltage (C, bottom left). Notably, the Jacobian, and thus the IV estimate, was proportional to the conductance matrix (W_0) of our model (C, bottom right). This can be explained by the similar average voltages and identical reversal potentials for neurons, such that Supplementary Information Equation 6 simply scales W_0.

When inputs were applied to induce widely varying average voltages across neurons (Extended Data Fig. 3D), the IV estimator still converged to the Jacobian. However, the relationship to W_0 was weak because the reversal potentials and the slope of the non-linearity were different for each neuron, thus rescaling each entry of W_0 in Supplementary Information Equation 6.

In this latter case, the utility of the connectome prior may be questioned—the relationship between number of synapses (a proxy for total conductance) to the Jacobian, the IV estimand, will be corrupted. Nevertheless, a critical correspondence between the Jacobian and conductance remains: if $W_{0_{i,j}} = 0$ then $J_{i,j} = 0$, which can be deduced from Supplementary Information Equation 6. We demonstrated this retained benefit in a simulation with sparse connectivity (Extended Data Fig. 4), where IV-Bayes converged far more rapidly to the Jacobian than the naive IV approach (C).

In our simulations, we considered neural dynamics that are only slightly perturbed about a steady state for an extended period. However, in most naturalistic settings, this level of stability is unlikely. During naturalistic behavior an animal will display large variations in neural activity. Under such conditions, the IV estimate would be a mixture of potentially diverse Jacobians and thus may not accurately describe the underlying neural dynamics.

Thus, to enhance the interpretability and efficiency of estimating IV, it is crucial to minimize uncontrolled variability in membrane voltage. A general strategy can be to linearize around a voltage trajectory instead of a fixed voltage. For instance, in a sensory stimulation context, the peri-stimulus average voltage traces from randomized stimulation events can serve as an estimated trajectory. The IV estimate can then be computed as a function of time relative to the stimulus onset. If neuronal variability is sufficiently reduced or 'quenched' [59], the estimated time varying Jacobian will be accurate as a linear approximation. More broadly, conditioning the IV estimate on randomized experimental interventions or external covariates (e.g., behavior) that are independent of optogenetic perturbation will reduce the conditional variance of neural dynamics (i.e., $\mathrm{Var}[Y] \geq \mathbb{E}[\mathrm{Var}[Y|X]]$).

We have analyzed the properties of the IV estimator in the context of a conductance based neural dynamics simulation. It is important to note that we have hand tuned the properties of these simulations such that our estimator is efficient and neural dynamics are stable. We note that a condition under which estimation became intractable was when the neuronal time constant was large relative to the time scale of stimulation. The effective low pass filtering made downstream effects of white noise stimulation difficult to detect. Ameliorating this by increasing the strength of stimulation would eventually push the simulation into chaotic regimes. Yet the time scale of stimulation,

in our estimation scheme, cannot go below conductance delays, which are often short. To address this it will be important to consider extensions to techniques that can account for temporal correlation in the instrument [29].

IV estimator additional simulations

The IV-Bayes estimator will recover a ground truth effectome even if the connectome prior is independent of the ground truth. Yet, a misspecified prior will reduce the efficiency of the estimator inversely proportional to the prior variance. With low variance on a misspecified prior it will take a larger amount of data to outweigh the prior. We ran a small simulation to demonstrate this by varying the magnitude of the constant we add to the prior variance (see caption Fig. 2C), simulating a sparse correct effectome, and sparse incorrect effectome (Extended Data Fig. 5A), then applied IV Bayes with the correct and incorrect effectome as prior means (B).

We find that when prior variance is small (B left) the estimator with the correct prior (blue) converges more rapidly than the naive estimate with no prior (green) and far faster than the estimator with the incorrect prior (orange). As the prior variance increases (B left to right) the estimator with the incorrect prior becomes more efficient while the estimator with the correct prior becomes less efficient.

We also considered the case where we apply our AR(1) estimator to slower time scale interactions (AR(P), Extended Data Fig. 6A). We find that it remains a consistent estimator of the first order parameters of the AR(P) process but that the convergence is slower (B, blue above orange trace).

Additional connectome analyses

We focused our additional analyses on the unique eigencircuits associated with the first 1,000 eigenvalues (516 because complex eigenvalues come in conjugate pairs). We analyzed the distribution of time scales, localization of synapses, synapse count, and sign. We find that the fastest time scales tend to be disproportionately in the earliest modes and slower time scales in lower modes (Extended Data Fig. 8B). We interpret this to imply that the largest dynamical modes in the fly reflect reciprocal inhibition instantiated with large numbers of synapses. Most eigencircuits have rotational dynamics at intermediate time scales (B). In general eigencircuits tend to have similar numbers of excitatory and inhibitory synapses but when there is an imbalance synapses tend to be predominantly inhibitory (C). There was a tendency for the fastest time scales to have majority inhibitory weights (D). Most eigencircuits were not localized but localized circuits could be found up to the 200th eigencircuit (E,F) and there were certainly dominating non-local circuits (e.g., eigencircuits 8, 10, and 12).

We were surprised at how our eigencircuit analysis indicated whole fly brain dynamics were high-dimensional—in contrast to many findings on the low dimensional nature of neural dynamics. We wondered how this low-dimensionality related to connectivity. Cell-type and region-based analyses are excellent directions for future research that would require careful thought about the form of the relationship between cell-types and regions to effects between neurons.

To preliminarily address these questions we performed several global transformations of our original connectome and found the dimensionality result was quite robust to them (Extended Data Fig. 10). We applied a hyperbolic tangent nonlinearity and found dimensionality increased only slightly–even when only the sign of the connectome was preserved. Adding measurement error noise also did not change the dimensionality.

We considered whether the dimensionality might be a simple consequence of the high sparsity of the connectome. To test this we shuffled the synaptic weights of the connectome, corrupting patterns of connectivity while preserving the same marginal sparsity and found that the dimensionality was greatly increased (dotted lines). This suggests that dimensionality is less likely to be influenced by any global changes but instead by specific alterations to patterns of connectivity.

More generally, the robustness of eigencircuits is an important question: would our results change if the protocol for measuring the connectome had changed, or if the connectome of a different fly had been measured? We preliminarily addressed this by sampling noisy connectomes to model both biological variability (across animals) and measurement error (missed or miscounted synapse numbers). We do so by computing the eigencircuits of the connectome plus gaussian noise proportional to the synaptic count (a linear mean variance relationship, common in counting distributions). We then measured all pairwise correlations between the top 250 eigencircuits between the original and corrupted connectome (removing redundant conjugate pairs). We then plotted the max absolute r-value for each original eigencircuit. For the eigencircuits with only real parts this was a straightforward calculation of r-values. For the complex eigencircuits (with real and imaginary coefficients) we regressed the real and imaginary components onto those of another then calculated the r-value of this linear fit. We find that in general for higher SNR 10-100 the top eigencircuits are nearly identical (Extended Data Fig. 9A, black, grey traces near 1). For lower SNR's most of the top eigenvectors are still recovered but a few are lost (red trace below $|r| \approx 0.5$).

Typically, neuroscientists have studied anatomically localized neural circuits in the fly. We were intrigued by the putative dominant dynamical modes that spanned multiple neuropil but sought to determine if they were as robust as the localized circuits to help motivate their further study. To determine if non-local eigencircuits were robust relative to the more localized circuits we examined the correlation between the localization index with the degree of corruption from the highest noise added to the connectome condition above (Extended Data Fig. 9A red). We found a significant correlation where more localized circuits were more robust to added noise. Yet there were many examples of non-localized circuits that were robust to noise (B).

We performed the eigendecompisition of the matrix of synaptic counts and signs. It is unclear whether this the optimal linear approximation to nonlinear neuronal dynamics of the fly. To preliminarily address the robustness of our results to this choice we considered a nonlinear transform of synaptic weights. We used a saturating function (hyperbolic tangent) following the intuition that the magnitude of effects between neurons will eventually saturate as postsynaptic voltage nears the synaptic reversal potential (a fact not accounted for by classic firing rate models of neural dynamics). We titrated the magnitude of this effect by rescaling the connectome weights relative

to the max count of synapses (Extended Data Fig. 9C). In general we found a gradual degradation of the original eigencircuits. Surprisingly, some eigencircuits were even retained after only the sign of the connectome was preserved (D, green trace above 0.5).

Our eigencircuit analysis also revealed the presence of non-localized circuits that became more prevalent with increasing eigenvalue rank. A sample of these non-localized circuits reveals diverse innervation patterns (Extended Data Fig. 7). Eigenvector 59 contains neurons that innervate both optic lobes and the central complex (Extended Data Fig. 7, top). This circuit has a real negative eigenvalue, indicating inhibition under rectified dynamics, which may permit selective integration of visual input from a single eye depending on the strength of visual input. We also found circuits that involve multiple sensory modalities, such as eigenvector 67 (Extended Data Fig. 7, middle), which contains visual neurons in the optic lobe (not included in the render due to our visualization threshold: see Results, 'Putative global dynamical properties of the fly central nervous system') and olfactory neurons in the antennal lobe and lateral horn. This circuit has a complex eigenvalue, which suggests oscillatory dynamics. Interestingly, the loadings for this eigenvector have different signs (data not shown), indicating that these neurons oscillate out of sync. Finally, we consider eigenvector 76, with neurons primarily located in visual and pre-motor areas (Extended Data Fig. 7, bottom). This circuit is also associated with a complex eigenvalue, suggesting oscillatory dynamics. Given this circuit's incorporation of both visual neurons and descending output neurons, we speculate that this circuit may be involved in coordinating visually-driven motor programs.

We also investigated the robustness of these non-localized eigencircuits to different choices in the number of neurons to include for each circuit (Extended Data Fig. 9E). First, we examined the robustness of our localization index, which calculates the fraction of neurons in the most highly represented neuropils relative to the total number of neurons in the circuit. We adjusted the loading threshold and the number of top neuropils to include (E, left) and found that the pattern of localization vs. rank is consistent: dominant eigenvectors tend to be localized, while less dominant eigenvectors tend to be widely distributed across the brain. To further validate this finding, we examined the relationship between number of neurons in each eigenvector and the loading threshold (E, right). Once again, the relationship is consistent across inclusion criteria: dominant eigenvectors are sparse, while less dominant eigenvectors contain many neurons. Together, these results indicate that non-localized eigencircuits are a robust feature of the putative effectome.

References

[46] Adesnik, H. & Abdeladim, L. Probing Neural Codes with Two-Photon Holographic Optogenetics. *Nature Neuroscience* 24, 1356–1366 (2021).

[47] Aragon, M. J. et al. Multiphoton imaging of neural structure and activity in Drosophila through the intact cuticle. *eLife* 11, e69094 (2022).

[48] Isaacman-Beck, J. et al. SPARC enables genetic manipulation of precise proportions of cells. *Nature Neuroscience* 23, 1168–1175 (2020).

[49] Kadas, D., Duch, C. & Consoulas, C. Postnatal Increases in Axonal Conduction Velocity of an Identified Drosophila Interneuron Require Fast Sodium, L-Type Calcium and Shaker Potassium Channels. *eNeuro* 6, ENEURO.0181–19.2019 (2019).

[50] Zhang, T. et al. Kilohertz two-photon brain imaging in awake mice. *Nature Methods* 16, 1119–1122 (2019).

[51] Chamberland, S. et al. Fast two-photon imaging of subcellular voltage dynamics in neuronal tissue with genetically encoded indicators. *eLife* 6, e25690 (2017).

[52] Yang, H. H. et al. Subcellular imaging of voltage and calcium signals reveals neural processing in vivo. *Cell* 166, 245–257 (2016).

[53] Marshel, J. H. et al. Cortical layer–specific critical dynamics triggering perception. *Science* 365, eaaw5202 (2019).

[54] Klapoetke, N. C. et al. Independent optical excitation of distinct neural populations. *Nature Methods* 11, 338–346 (2014).

[55] Brezovec, L. E. et al. BIFROST: A method for registering diverse imaging datasets. (2023) doi:10.1101/2023.06.09.544408.

[56] Avants, B. B., Tustison, N., Song, G., et al. Advanced normalization tools (ANTS). *Insight J* 2, 1–35 (2009).

[57] Hoffmann, M. et al. SynthMorph: Learning contrast-invariant registration without acquired images. *IEEE Transactions on Medical Imaging* 41, 543–558 (2021).

[58] Costa, M., Manton, J. D., Ostrovsky, A. D., Prohaska, S. & Jefferis, G. S. NBLAST: Rapid, sensitive comparison of neuronal structure and construction of neuron family databases. *Neuron* 91, 293–311 (2016).

[59] Churchland, M. M. et al. Stimulus onset quenches neural variability: A widespread cortical phenomenon. *Nature Neuroscience* 13, 369–378 (2010).
